# Noninvasive Bimodal Neuromodulation for the Treatment of Tinnitus: Protocol for a Second Large-Scale Double-Blind Randomized Clinical Trial to Optimize Stimulation Parameters

**DOI:** 10.2196/13176

**Published:** 2019-09-27

**Authors:** Brendan Conlon, Caroline Hamilton, Stephen Hughes, Emma Meade, Deborah A Hall, Sven Vanneste, Berthold Langguth, Hubert H Lim

**Affiliations:** 1 Department of Otolaryngology St James Hospital Dublin and Tallaght University Hospital Dublin Dublin Ireland; 2 Neuromod Devices Limited Dublin Ireland; 3 Trinity College Dublin Dublin Ireland; 4 Hearing Sciences Division of Clinical Neuroscience University of Nottingham Nottingham United Kingdom; 5 National Institute for Health Research Nottingham Biomedical Research Centre Nottingham United Kingdom; 6 University of Nottingham Malaysia Semenyih, Selangor Darul Ehsan Malaysia; 7 University of Texas at Dallas Richardson, TX United States; 8 Department of Psychiatry and Psychotherapy, University of Regensburg Regensburg Germany; 9 University of Minnesota Minneapolis, MN United States

**Keywords:** tinnitus, bimodal, neuromodulation, trigeminal nerve, vagus nerve, auditory nerve, auditory cortex, pain, plasticity, precision medicine

## Abstract

**Background:**

There is increasing evidence from animal and human studies that bimodal neuromodulation combining sound and electrical somatosensory stimulation of the tongue can induce extensive brain changes and treat tinnitus.

**Objective:**

The main objectives of the proposed clinical study are to confirm the efficacy, safety, and tolerability of treatment demonstrated in a previous large-scale study of bimodal auditory and trigeminal nerve (tongue) stimulation (Treatment Evaluation of Neuromodulation for Tinnitus - Stage A1); evaluate the therapeutic effects of adjusting stimulation parameters over time; and determine the contribution of different features of bimodal stimulation in improving tinnitus outcomes.

**Methods:**

This study will be a prospective, randomized, double-blind, parallel-arm, comparative clinical trial of a 12-week treatment for tinnitus using a Conformité Européenne (CE)–marked device with a pre-post and 12-month follow-up design. Four treatment arms will be investigated, in which each arm consists of two different stimulation settings, with the first setting presented during the first 6 weeks and the second setting presented during the next 6 weeks of treatment. The study will enroll 192 participants, split in a ratio of 80:80:16:16 across the four arms. Participants will be randomized to one of four arms and stratified to minimize baseline variability in four categories: two separate strata for sound level tolerance (using loudness discomfort level as indicators for hyperacusis severity), high tinnitus symptom severity based on the Tinnitus Handicap Inventory (THI), and tinnitus laterality. The primary efficacy endpoints are within-arm changes in THI and Tinnitus Functional Index as well as between-arm changes in THI after 6 weeks of treatment for the full cohort and two subgroups of tinnitus participants (ie, one hyperacusis subgroup and a high tinnitus symptom severity subgroup). Additional efficacy endpoints include within-arm or between-arm changes in THI after 6 or 12 weeks of treatment and in different subgroups of tinnitus participants as well as at posttreatment assessments at 6 weeks, 6 months, and 12 months. Treatment safety, attrition rates, and compliance rates will also be assessed and reported.

**Results:**

This study protocol was approved by the Tallaght University Hospital/St. James’s Hospital Joint Research Ethics Committee in Dublin, Ireland. The first participant was enrolled on March 20, 2018. The data collection and database lock are expected to be completed by February 2020, and the data analysis and manuscript submission are expected to be conducted in autumn of 2020.

**Conclusions:**

The findings of this study will be disseminated to relevant research, clinical, and health services and patient communities through publications in peer-reviewed journals and presentations at scientific and clinical conferences.

**Trial Registration:**

ClinicalTrials.gov NCT03530306; https://clinicaltrials.gov/ct2/show/NCT03530306

**International Registered Report Identifier (IRRID):**

DERR1-10.2196/13176

## Introduction

Tinnitus is the perception of sound in the absence of an external auditory stimulus and is commonly described as “ringing in the ears.” The condition significantly affects approximately 5%-10% of the global population [1-3]. Tinnitus is heterogeneous, with a diverse range of etiologies, but is believed to be commonly accompanied by a sensorineural hearing loss [4-6]. One ongoing hypothesis is that the decreased input into the peripheral auditory system due to hearing loss causes spatial reorganization of the brain or compensatory changes in firing activity in multiple regions along the ascending auditory and nonauditory pathways that can lead to the tinnitus percept [3,5,7,8].

In individuals with normal hearing, sound travels as vibrations through the outer and middle ears into the cochlea, where cells within the cochlea convert the vibrations into neural signals that are transmitted along the auditory nerve to the brain [9,10]. The neural signals travel up through the brainstem, midbrain, and thalamus to the auditory cortex for sound perception. The ascending auditory pathway has a well-organized spatial map of frequencies (ie, neurons located in a certain region respond best to a specific sound frequency, and this spatial ordering of frequencies is known as tonotopy or a tonotopic map). In addition to the ascending pathway, there are dense descending connections from higher auditory and cognitive centers down to earlier stages of auditory neurons, which provide a way for sound perception to be modified or fine-tuned by attention and learning centers [11-16]. Furthermore, there are widespread projections from limbic and nonauditory pathways, such as somatosensory pathways, to the auditory network [17-27].

In tinnitus patients, the abnormal reorganization of the auditory brain can occur as spatial reorganization of the tonotopic map or changes in neural firing in one or several of the auditory regions [3,5,7]. For example, a high-frequency hearing loss could lead to a downregulation of peripheral synapses and activity in the high-frequency region of the thalamus (eg, medial geniculate body) and auditory cortex, in which those neurons then become more sensitive and active to lower frequency sounds (ie, an expanded frequency representation in the auditory brain for lower frequencies). Due to this frequency expansion and changes in firing patterns in those regions (eg, hyperactivity or hypersynchrony across neurons), the patient experiences a phantom percept (tinnitus) corresponding to that expanded brain region. There are recent studies suggesting that topographic reorganization may not be necessary for tinnitus or phantom sensations, in general [28,29]. It may be possible that the central auditory system broadly overcompensates for the loss of peripheral input and increases the central gain in different networks of neurons along the ascending auditory pathway. In connection with multiple nonauditory brain regions, this enhanced gain across the auditory network may not only cause excessive cortical activity and phantom sound awareness, but can also link and worsen the emotional and cognitive/memory attributes with the phantom percept [30,31].

The most commonly used approach for treating tinnitus is auditory stimulation, such as sound amplification (eg, hearing aids) or sound therapy (eg, noise maskers, tone sequences, or music therapy), which are intended to drive additional input into the auditory system and interact with the abnormal auditory neurons involved with tinnitus [32-36]. Based on extensive research in animals and several human studies, an emerging approach for driving strong plasticity and altering neurons within the auditory system is bimodal neuromodulation using acoustic stimulation combined with a nonauditory input, such as with vagus, somatosensory, or trigeminal nerve stimulation [20,37-45]. Since somatosensory or trigeminal inputs can activate or modulate neurons throughout the auditory pathway [20,23,24,27,46-52], combining sound stimulation with electrical stimulation of different body locations, especially via cranial nerves, has gained increasing interest as a promising approach for reversing the abnormal patterns of auditory neurons associated with tinnitus. Relevant to the proposed clinical study, experiments in animals have shown that combining sound stimulation with electrical stimulation of the tongue can drive extensive changes across the auditory system up to the midbrain and cortex that can potentially treat tinnitus, in which electrical stimulation of the tongue could drive greater auditory plasticity than stimulation of other somatosensory or trigeminal inputs [20].

To date, there have only been a limited number of small and uncontrolled pilot studies to assess the safety and efficacy of bimodal neuromodulation approaches employing sound stimulation combined with cranial nerve stimulation for tinnitus treatment. These include invasive vagus nerve stimulation [41,53], noninvasive stimulation of the vagus nerve [54-56], and noninvasive cervical or trigeminal nerve stimulation [42-44,46,47]. Although the vagus nerve stimulation demonstrated promising results in animals [37], human studies have shown mixed results [41,53]. Published human studies using noninvasive cervical or trigeminal nerve stimulation have demonstrated promising initial efficacy [42-44]. However, these results should be considered preliminary, as the data stem from small pilot studies. Therefore, progression to properly designed, sufficiently powered, blinded, randomized clinical trials are critically needed in the tinnitus field [34,36,57,58] to further confirm the efficacy and safety of bimodal neuromodulation combining sound and cranial nerve stimulation.

**Figure 1:**
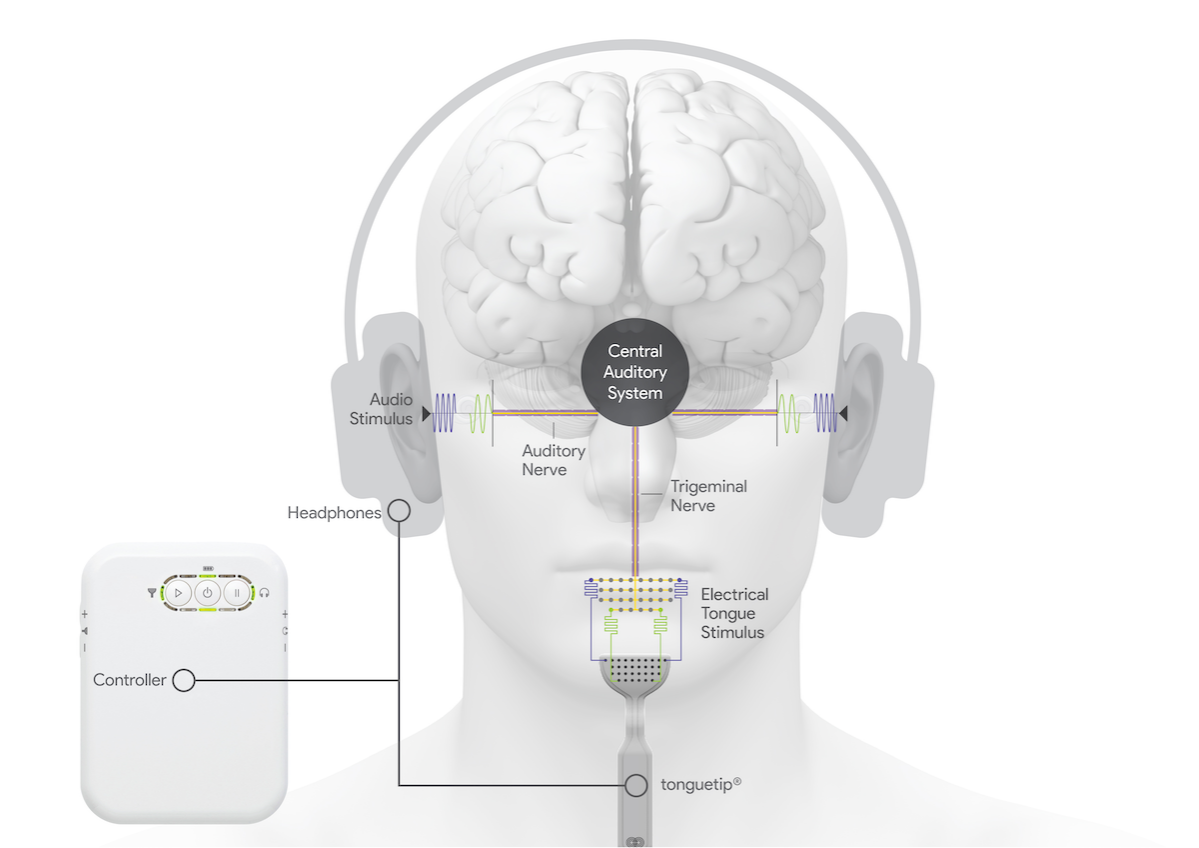
Bimodal sensory neuromodulation device (Lenire) for tinnitus treatment. The system developed by Neuromod Devices (Dublin, Ireland) consists of wireless high-fidelity circumaural headphones that deliver acoustic stimuli, a 32-site surface electrode array (tonguetip) for presenting electrical stimulus patterns to the anterior dorsal surface of the tongue, and a battery-powered controller that coordinates both stimulus modalities.

This study protocol is part of a major clinical development program sponsored by Neuromod Devices (Dublin, Ireland) to provide large-scale clinical evidence of the safety and efficacy of a new bimodal neuromodulation treatment for tinnitus (using acoustic and trigeminal nerve stimulation; Figure 1). This study protocol is designed to confirm and potentially enhance, through further stimulation optimization, the clinical efficacy demonstrated in a recently completed clinical trial (Treatment Evaluation of Neuromodulation for Tinnitus - Stage A1 [TENT-A1]) that evaluated bimodal neuromodulation in 326 tinnitus participants. The TENT-A1 protocol has been previously published [59]. TENT-A1 was a double-blind, two-site randomized study that evaluated the relative efficacy and safety of three different settings for acoustic and trigeminal stimulation (ie, settings related to acoustic frequencies and background noise, electrical stimulation patterns on the tongue with a 32-site surface electrode array, and intermodality delays). The treatment period was 12 weeks, wherein the therapeutic effects were assessed during treatment and at several follow-up visits up to 12 months posttreatment. Participants were presented with one stimulation setting for the entire 12-week treatment period. The positive results from TENT-A1 [60] have led to further questions and new directions for confirming and further optimizing stimulation parameters for bimodal neuromodulation, which will be investigated through the protocol presented in this paper describing a follow-up, double-blind, randomized clinical trial (Treatment Evaluation of Neuromodulation for Tinnitus - Stage A2 [TENT-A2]) in 192 participants with tinnitus.

The primary objectives of TENT-A2 are to (1) confirm the positive therapeutic effects, safety profile, and tolerability of treatment observed in TENT-A1; (2) determine the therapeutic effects of changing the stimulation parameters over time, in which the first stimulation setting is presented during the first 6 weeks of treatment and a second stimulation setting is presented during the next 6 weeks of treatment; and (3) assess how treatment outcome depends on the contribution of different acoustic or tongue stimuli not tested in TENT-A1. TENT-A2 also investigates the relative response of patient subtypes to the different treatment parameters. Building on the data collected in TENT-A1, this study will allow for the continued collection and analysis of safety data.

## Methods

### Trial Design

TENT-A2 is a prospective, single-site, parallel-arm, randomized, double-blind, comparative study investigating the safety and efficacy of four different treatment arms. The treatment will be evaluated for 12 weeks, during which different parameter settings will be delivered sequentially in the first and second 6-week segments of treatment (Table 1). Participant assessments will be performed at screening, enrollment (start of treatment), interim (after 6 weeks of treatment with the first stimulation setting), and end of treatment (after 6 weeks of treatment with the second stimulation setting). Posttreatment assessments will be conducted at the 6-week follow-up, 6-month follow-up, and 12-month follow-up (Table 2). TENT-A2 will be conducted at the Wellcome Trust-HRB Clinical Research Facility at St. James’s Hospital in Dublin, Ireland. The protocol was independently reviewed and approved by Research Ethics Committees of the Tallaght University Hospital - St James’s Hospital (Reference: 2018-03-List 9). The trial is sponsored by Neuromod Devices. Our reporting follows standard protocol items for clinical trials defined in the SPIRIT 2013 Statement [61].

**Table 1:** Stimulation parameter settings that will be utilized for the four parallel treatment arms of the TENT-A2 study. Two different stimulus settings will be used for each treatment arm during the first and second 6-week periods of the 12-week treatment. Parameter setting (PS) labels listed in the table are specific names used internally in the company. PS1 is a stimulus setting equivalent to the one used in the previous TENT-A1 study to assess repeatability of results between two different studies. PS1 consists of a sequence of tones mixed with structured wideband noise, in which the tones are synchronized in time with electrical pulses presented to the tongue (for further details, see published protocol paper for TENT-A1 [59]). One or more acoustic or electrical features in PS1 are modified or removed to create the other proprietary stimulus settings used in the TENT-A2 study. A general description of the different stimuli is included in the table to sufficiently understand the rationale of the study design but without fully revealing the specific stimulation algorithm for each arm.

Treatment	First 6 weeks	Next 6 weeks
Arm 1	PS1^a^: Same stimulation setting used in Arm 1 of TENT-A1^b^ for comparison of findings with TENT-A2^c^. A wide range of pure tones (0.5-7 kHz) are presented binaurally with each tone synchronized in time with an electrical pulse train that is presented to specific locations on the tongue via the 32-site tonguetip component. A background wideband noise is also mixed in with the stimuli. Presentation rate of each paired stimuli is approximately 12.5 Hz.	PS4: Similar to PS1, except that a randomly varying short delay (0-30 ms) is introduced between the tone and tongue stimuli, and the location of stimulation on the tongue is randomized across stimuli. PS4 is designed to investigate if a different stimulation setting from PS1 can drive additional therapeutic effects beyond the plateau effects observed for PS1 in TENT-A1.
Arm 2	PS6: Low-frequency pure tones (0.5-1 kHz) are presented binaurally with randomly varying long delays (~1 s) between each tone and tongue stimuli, in which the location of stimulation on the tongue is randomized across stimuli. Background noise is not included in PS6. Presentation rate is approximately 0.5 Hz. PS6 is designed to determine if specific features of PS1 are required for improvements in tinnitus symptom severity, in which TENT-A2 is powered to detect a clinically meaningful difference between PS1 and PS6.	PS10: Similar to PS4, with the main difference involving the use of a wideband noise instead of pure tones for the sound stimulus. PS10 is designed to investigate the efficacy and tolerability of tongue stimulation with a noise stimulus instead of pure tones.
Arm 3	PS7: Similar to PS6, except that the sound stimuli consisted of multiple simultaneous tones instead of single tones. PS7 is designed to investigate if broader spectrum tonal stimuli can drive additional therapeutic effects compared to PS6.	PS4: See description for PS4 above. PS4 is introduced in Arm 3 to allow for comparison with PS4 in Arm 1 and to assess if different stimulation settings during the first 6 weeks affect the therapeutic effects observed during the next 6 weeks.
Arm 4	PS9: Acoustic-only condition with same stimuli as PS6 but without tongue stimulation.	PS6: See description for PS6 above. The tonguetip is provided for tongue stimulation to investigate the therapeutic effects of bimodal stimulation compared to acoustic-only stimulation and to provide participants with a stimulation setting that is expected to improve tinnitus during the 12-week treatment period based on findings from TENT-A1. Therefore, participants in Arm 4, as with the other arms, are informed that they are randomly allocated to a treatment arm to maintain blinding (ie, all participants know they are receiving treatment and they do not know which treatment arm is supposed to include the most effective settings).

^a^PS: parameter setting.

^b^TENT-A1: Treatment Evaluation of Neuromodulation for Tinnitus - Stage A1.

^c^TENT-A2: Treatment Evaluation of Neuromodulation for Tinnitus - Stage A2.

**Table 2:** Schedule of visits, tasks, and assessments for TENT-A2 study.

TENT-A2^a^ timeline		Screening	Enrollment and fitting	Post-allocation					Follow-up		
				Telephone call	Interim visit	Telephone call	Endpoint visit				
		t – 8 wk	t = 0 wk	t + 3 wk	t + 6 wk	t + 9 wk	t + 12 wk	t + 18 wk		t + 38 wk	t + 64 wk
**Task**											
	Eligibility screen	✓									
	Informed consent	✓									
	Allocation		✓								
	Training on using the device		✓								
	Review of device usage data				✓						
	Encourage subject compliance		✓	✓	✓	✓					
	Return device						✓				
**Intervention**											
	Arm 1		✓	✓	✓	✓	✓				
	Arm 2		✓	✓	✓	✓	✓				
	Arm 3		✓	✓	✓	✓	✓				
	Arm 4		✓	✓	✓	✓	✓				
**Assessment**											
	Medical history	✓					✓				
	Medications or illnesses	✓	✓		✓		✓	✓		✓	✓
	Audiometric test of hearing	✓					✓				
	Tinnitus location and tonality	✓	✓		✓		✓	✓		✓	✓
	Tinnitus loudness matching	✓									
	Loudness discomfort level	✓	✓		✓		✓	✓		✓	✓
	Mini-Mental State Examination	✓									
	State-Trait Anxiety Inventory	✓									
	Somatic assessment		✓								
	Oral assessment		✓				✓				
	Minimum masking level	✓	✓		✓		✓	✓		✓	✓
	Pittsburgh Sleep Quality Index	✓	✓		✓		✓	✓		✓	✓
	Tinnitus Handicap Inventory	✓	✓		✓		✓	✓		✓	✓
	Tinnitus Functional Index	✓	✓		✓		✓	✓		✓	✓
	Visual analogue scales	✓	✓		✓		✓	✓		✓	✓
	Hyperacusis questionnaire		✓		✓		✓	✓		✓	✓
	Clinical Global Impression				✓		✓	✓		✓	✓
	Adverse Events	✓	✓		✓		✓	✓		✓	✓
	Device usability questionnaire						✓				
	Demographic data							✓			

^a^TENT-A2: Treatment Evaluation of Neuromodulation for Tinnitus - Stage A2.

^b^t: timepoint from enrollment and fitting.

### Eligibility Criteria

Eligible participants will be aged 18-70 years at screening, self-report experiencing predominantly tonal tinnitus for >3 months and <10 years, score 38-100 points on the Tinnitus Handicap Inventory (THI), have a wide-band noise Minimum Masking Level (MML) measurement between 20 and 80 dB hearing level (HL), be able to read and understand English, be willing and able to provide informed consent, and be willing to commit to the full duration of the study. Participants with predominantly tonal tinnitus (not atonal tinnitus) will be recruited to simplify the study design and analyses to a less diverse and heterogeneous group. In the TENT-A1 study, participants with tinnitus lasting for 3 months up to 5 years were recruited. The same lower boundary will be used in the TENT-A2 study, in which the Sponsor and its Scientific Advisory Committee originally considered 3 months as the start of transition from acute to chronic tinnitus. The upper boundary was increased to 10 years to enhance recruitment within a shorter period of time than that in the TENT-A1 study.

Candidates will be excluded if they have objective tinnitus; pulsatile tinnitus (rhythmical sounds that often beat in time with the heartbeat); somatic tinnitus caused by a head or neck injury; or tinnitus that is comorbid with a neurological condition that may lead to loss of consciousness or is considered to be the dominant feature of the tinnitus, as assessed by an audiologist or clinician. Abnormal otoscopy or abnormal tympanometry, as possible indicators of conductive hearing loss, are exclusion criteria, as is a sensorineural hearing loss either unilaterally or bilaterally, wherein the subject has >40 dB HL in at least one measurement frequency in the range of 0.25-1.00 kHz or has >80 dB HL in at least one measurement frequency in the range of 2.0-8.0 kHz. In addition, we will also exclude participants who began wearing a hearing aid within 90 days prior to eligibility assessment, those with any type of electroactive implantable device (eg, vagal nerve stimulator, cochlear implant, or a cardiac pacemaker) and those with the following conditions that can be comorbid with tinnitus: Meniere’s disease, loudness discomfort level for sounds presented <30 dB sensation level, temporomandibular joint disorder, and psychological conditions determined by a score >120/160 on the State-Trait Anxiety Inventory (STAI) [62,63]. Moderate to severe dementia, as indicated by a score <20 on the Mini-Mental State Examination (MMSE) [64], will also be a sufficient reason for exclusion. A final set of exclusion criteria based on medical history taken at the screening assessment include oral piercings, pregnancy, involvement in medicolegal cases, history of auditory hallucinations, current prescription of a drug for a central nervous system pathology (ie, epilepsy, multiple sclerosis, Parkinson disease, and bipolar disorder), and previous use of a Neuromod Devices product. Finally, the participant may be excluded if the principal investigator does not deem the candidate suitable for the study for reasons not listed above.

### Intervention

Participants enrolled in the trial will be given a proprietary Conformité Européenne (CE)-marked Class IIa medical device, which comprises bimodal auditory and trigeminal nerve (tongue) stimulation from the sponsor company (Figure 1; Neuromod Devices, Dublin, Ireland). High-fidelity Bluetooth headphones deliver the auditory stimulation, which includes sequences of pure tones and wideband noise. The trigeminal nerve is stimulated electrically via a 32-electrode transmucosal array placed on the anterior dorsal surface of the tongue. Tongue stimulation is delivered in the form of biphasic anodic-leading pulses of duration between 5 and 130 μs and fixed amplitude. The electrodes in the array are stimulated in a temporospatial pattern that represent features of the acoustic stimulus, such as the frequencies and onset of stimulus tones. Each stimulation parameter setting (PS) listed in Table 1 represents a different combination of acoustic and tongue stimulation patterns or delays that are being evaluated in this study. For example, PS1 and PS6 are used in the first 6 weeks of Arms 1 and 2, respectively, to allow comparison of an effective treatment setting from the TENT-A1 study (ie, PS1) with another bimodal condition (ie, PS6) to identify specific bimodal stimulation features for improving tinnitus symptom severity. The TENT-A2 study has been powered to detect a clinically meaningful difference between PS1 and PS6 during the first 6-week period. Additional stimuli listed in Table 1 and comparisons described in the Statistical Methods section have been included to achieve the main objectives of this TENT-A2 study. Further details and rationale for the different stimulation settings used within each treatment arm are provided in Table 1. Note that the current CE-marked 32-site electrode array (the tonguetip shown in Figure 1) has been successfully used for stimulation of the tongue to improve tinnitus symptom severity in the TENT-A1 study [60] and is used in this TENT-A2 study to further investigate the contribution of different stimulus features on therapeutic outcomes, as described in Table 1. In future studies, the minimum number of electrodes on the tongue required for sufficient therapeutic effects can be investigated.

Each participant’s pure-tone audiometric thresholds (in the range 0.25 to 8 kHz) will be captured at the screening visit and subsequently used to configure the intensity of the auditory stimuli, typically 10 dB sensation level or more above their hearing thresholds. The participant will be provided with an option to adjust the default auditory stimulus intensities from –12 dB to +12 dB in 2-dB increments during treatment. For safety reasons, the upper level of stimulus intensity is limited for participants with >70 dB HL hearing loss at any frequency. The treatment device reverts to the default stimulus intensities at the start of each new treatment session. Any adjustments made by the participants to the stimulus intensities are logged in the device’s memory for subsequent analysis.

The tongue stimulus intensity will be configured for each participant at enrollment, based on a calibration procedure that determines the participant’s threshold of perception and sets the intensity at a suprathreshold and comfortable level. During treatment, the participant is also provided with the option to adjust the tongue stimulus intensity up to a maximum of 60% above the calibrated level or down to a minimum of 40% below the calibrated level, to allow participants to adjust for natural variances in somatosensory or perceptual sensitivity (eg, due to variations in electrolyte concentrations in the saliva or relative dryness in the mouth).

Participant usage and stimulus adjustments are logged automatically by the device, such as the time and date when the device is in use, the duration of electrode contact with the tongue, and the intensities of both the auditory and tongue stimuli.

Each device will be programmed with the personalized settings and treatment arm for each subject at the sponsor’s manufacturing site. The devices will be clearly identified with the participant’s unique identifier code (UIC). Investigators are extensively trained on fitting the device and instructing participants on its use per the manufacturer’s instructions. Participants will be provided with a training session on how to use the device at the enrollment visit. A *Quick Start Guide* and a *User Manual* will be provided to each participant to take home. Before leaving the clinical site at the enrollment visit, participants will complete a supervised treatment session that is at least 15 minutes in duration, to ensure that they are competent and comfortable using the device.

### Outcome Measures

Subjective clinical outcome measures commonly used to assess tinnitus symptom severity are the THI [65] and the Tinnitus Functional Index (TFI) [66,67]. The THI provides a measure of the emotional and functional impact of tinnitus, in which 25 items are scored 4/2/0 on a categorical scale corresponding to yes/sometimes/no, respectively. The global score of the THI has a value from 0 to 100, with a higher score indicating a greater negative impact of tinnitus. The TFI assesses a range of tinnitus-related functional complaints experienced over the week prior to assessment. Each of the 25 items is assessed on an 11-point Likert scale, and the sum of the scores is normalized to yield a global score of 0-100, with a higher score also indicating a greater negative impact. The Clinical Global Impression (CGI) is assessed at multiple visits to give an overall impression of the change in tinnitus (CGI-I) or sleep (CGI-S) since beginning treatment.

Tinnitus loudness is assessed by MML, tinnitus loudness matching (TLM), and a visual analogue scale (VAS). MML is a psychoacoustic estimate of the lowest level of wideband noise required to minimally mask the participant’s tinnitus [68]. The stimulus is presented binaurally, after the participant’s noise threshold level is obtained. TLM is assessed by presenting a 1-kHz tone contralateral to the predominant tinnitus ear or if tinnitus is equally loud in both sides or localized in the head, the stimuli are presented to the ear with better hearing [69]. The stimulus is increased until the participant confirms that it is equal in loudness to their tinnitus. TLM is only measured at screening. A VAS is employed for participants to rate the current loudness (or annoyance) of their tinnitus with 0 indicating “not loud at all” and 10 indicating “extremely loud” [70]. Both investigator-administered (MML and TLM) and participant-reported assessments (VAS) are used, because there is no agreed standard for assessing tinnitus loudness. Although a tinnitus loudness rating performs better against acceptability criteria for reliability and validity than a TLM or MML test, the rating question is limited because it is a single-item instrument and is probably able to detect only large changes [71].

Participant-reported and investigator-reported adverse events (AEs) will be recorded, classified, coded, and summarized. AEs will be classified according to severity, causality, and whether they are anticipated. They will be further coded by type for subsequent analysis, trending, and reporting purposes. Any treatment-related serious AEs will be reported to the local competent authority, the Research Ethics Committee, and the sponsor’s notified body, as required by local reporting regulations (in accordance with MEDDEV 2.12-1). The investigators will remain vigilant for signs of possible treatment-related changes in oral health (eg, irritation or discomfort in the oral cavity) and the impact on tinnitus.

Nonparticipant facing investigators will monitor the participants at the 6-week assessment, and the study may be stopped if the mean change in THI increases by 7 points and that in TFI increases by 13 points, or if the mean change in MML increases by 5.3 dB, in any treatment arm relative to enrollment values. Treatment-related changes in hearing thresholds that will be considered an AE is a deterioration from screening to the end of treatment of 15 dB in a minimum of two adjacent test frequencies (0.25-8 kHz) in either ear that cannot be explained by a conductive hearing problem or a recent excessive noise exposure and which continues at a subsequent follow-up visit. An additional safety endpoint will be that the mean change in hearing thresholds across all participants does not worsen by more than expected due to age-related hearing loss.

Compliance data will be extracted from log files on each participant’s device. The compliance rate will be expressed as a percentage of usage relative to the expected compliance as per the intended use for the device (a total of 42 hours over the 6-week period and a total of 84 hours over the 12-week period) and to a predefined minimum acceptable compliance threshold (defined as at least 3 hours of average usage within a 1-week period, corresponding to a sum total of 18 hours of treatment for the first 6-week period and 36 hours of treatment for the full 12-week period). This minimum acceptable compliance threshold is what was defined in the previous TENT-A1 study that still led to positive therapeutic effects for tinnitus treatment, and thus, a similar threshold is used in this study to enable comparison of results across studies.

### Recruitment

Participants will be recruited primarily via regional and national radio advertising that directs participants toward a dedicated trial sign-up website [72]. The recruitment website provides information on the study and how to proceed with registration. To register their interest, candidates must enter their email address, so that they can be emailed a UIC and personal identification number as well as a link to an online eligibility assessment (hosted by SurveyGizmo). To access the online eligibility assessment, candidates must click the link, which brings them to a log-in page that requires them to input their UIC and personal identification number. Once logged in, candidates can find further details about the requirements of participating in the study. Candidates will answer a set of general prescreening questions on age, duration of tinnitus, oral piercings, other current medical conditions, and other eligibility criteria-related questions. The online eligibility assessment is intended to reduce the burden of performing detailed screening visits on a large number of candidates who are expected to be interested in the trial, yet would not satisfy the inclusion and exclusion criteria. Candidates who meet the inclusion criteria will be provided with a participant information leaflet and informed consent form via email or post and invited to a screening visit at the Wellcome Trust-HRB Clinical Research Facility at St. James’s Hospital in Dublin, Ireland.

### Study Timeline

Participants will be expected to visit the clinic seven times throughout the entirety of the study. They will also receive two compliance telephone calls during the device usage period, one during the first 6 weeks and the other during the next 6 weeks of treatment. The schedule of clinical research activities is illustrated in Table 2. Various assessments will be completed by a multidisciplinary team including audiologists, medical doctors, physiotherapists, research nurses, and clinical investigators.

The screening visit will be used to determine whether a participant is eligible for enrollment into the trial, as defined by the abovementioned inclusion and exclusion criteria. The initial objective of the screening visit is to obtain written informed consent, in which the participants will be given sufficient time to read through the participant information leaflet and informed consent form. Initial outcome measure assessments, participant characteristics, and audiological profile are also obtained at the screening visit. This information is employed in the subgroup classification of participants, the stratified random allocation process, and for device configuration as described below.

At the enrollment (device fitting) visit, a physiotherapist will conduct a comprehensive assessment comprising a set of 25 predefined cranial manipulations designed to diagnose somatic tinnitus [26] as well as five additional maneuvers of the tongue. In this study, somatic tinnitus is defined as tinnitus where at least one of the somatic manipulations reliably produces a change in any psychoacoustic characteristics of a participant’s tinnitus (eg, in pitch, loudness, or localization). Assessments of outcome measures previously assessed at the screening visit are repeated at the enrollment visit. The enrollment visit also entails an oral health examination, device training and deployment, and a supervised treatment session. The treatment is self-administered by the participant daily for two 30-minute sessions over the course of the treatment. These sessions can be contiguous or completed at different times of the day.

The outcome measure assessments and safety information collection are repeated at the interim visit, halfway through the 12-week treatment. Compliance data will also be assessed and reviewed at the interim visit. Participants with poor compliance will be encouraged to improve their treatment device usage.

The assessments will be repeated at the endpoint visit (ie, end of 12-week treatment), including the outcome measure assessments and the oral health examination. An exit interview will be completed and the device will also be retrieved at the endpoint visit. Three follow-up visits will then be conducted to assess the posttreatment effects of the intervention. These posttreatment assessments will be conducted at the 6-week follow-up, 6-month follow-up, and 12-month follow-up, as listed in Table 2.

### Sample Size

Arm 1 and Arm 2 are powered to detect a between-arm clinically meaningful difference in the mean THI changes from enrollment to interim, where the clinically meaningful change in THI is considered to be 7 points [73]. The assumed sample standard deviation is 12 points, as estimated from a previous study sponsored by Neuromod Devices (TENT-A1) [60]. The sample size calculations were performed using Matlab 2016a (MathWorkds, Natick, Massachusetts), assuming a two-sided significance level of 0.025 (pairwise *t* test) and power of 90%, resulting in a total of 75 participants to be enrolled in treatment Arms 1 and 2. The remaining 0.025 of the overall 0.05 significance level is retained for within-arm and subgroup hypothesis tests.

Arms 3 and 4 are included for exploratory endpoints and are powered to detect a between-arm 10-point THI difference compared to Arm 1 from enrollment to interim. This requires approximately 15 participants in Arm 3 and Arm 4. Therefore, the allocation ratio among treatment arms is 5:5:1:1. In total, 180 participants (75+75+15+15) will be required to complete the interim assessment (first 6 weeks of treatment) across the four arms of the study. The attrition rate for the first 6 weeks of treatment in TENT-A1 was approximately 7%. Therefore, it is estimated that approximately 193 participants would need to be enrolled to ensure 180 participants complete the 6-week treatment assessment. This number is rounded down to 192 participants to ensure a balance at the required ratio (5:5:1:1 in Arms 1, 2, 3, and 4).

### Allocation

Eligible participants will be randomized as per the allocation ratio previously described (5:5:1:1) between the four parallel treatment arms (Table 1). Stratified randomization using the method of minimization [74] will be performed to balance the influence of several baseline covariates in the posthoc analyses. The stratification covariates are chosen based on the investigator’s research objective to elucidate relative treatment effects on possible subtypes of tinnitus participants with varying underlying characteristics. Allocation of participants will be stratified across the four intervention arms based on findings from TENT-A1 and in ranked order as per the following strata: (i) hyperacusis <70 dB sensation level at 500 Hz, (ii) hyperacusis <60 dB sensation level at 500 Hz (note that the loudness discomfort level assessment is used as an indicator for hyperacusis at screening), (iii) high THI of >56 points at screening, (iv) unilateral tinnitus as assessed at screening, and (v) participants who do not fall into the previous categories (note that this stratum will not be used to draw an inference).

### Data Collection

All data will be collected electronically using a validated electronic clinical case report form (eCRF) application. Participant data collected at all stages of the trial will be entered into the eCRF using UICs assigned to participants at recruitment phase. All participants and investigators performing the participant evaluations will be blinded to the allocation arm, and no allocation information will be contained in the eCRF. The data monitors will be able to remotely view the blinded data in the eCRF to monitor safety data.

### Statistical Methods

The primary efficacy analyses will focus on investigating: (1) within-arm (Arm 1) changes in THI and TFI from baseline (average of screening and enrollment) to interim (first 6 weeks of treatment) and (2) between-arm (Arm 1: Arm 2) changes in THI from enrollment to interim. These comparisons will be performed for the full cohort of participants as well as two subgroups of participants (ie, one hyperacusis subgroup and a high tinnitus symptom severity subgroup). The statistical analyses for testing these hypotheses while accounting for multiple comparisons are depicted in Figure 2, which shows how all primary efficacy analyses will be controlled at an overall significance level of 0.05 using a graphics-based sequential/parallel testing procedure with fallback [75]. The between-arm calculations use values from enrollment to interim in order to assess improvements in tinnitus symptom severity across different stimulation settings relative to the actual start of treatment. The within-arm calculations are using values from baseline to interim to match the design of the previous TENT-A1 study in order to allow direct comparison of findings across studies, which is one of the main objectives of this TENT-A2 study. The rationale for investigating the within-arm and between-arm changes for the hyperacusis subgroup (loudness discomfort level <70 dB sensation level at 500 Hz at screening) and the high tinnitus symptom severity subgroup (THI >56 points at screening), as well as stratifying the participants across treatment arms based on these subgroups, is that greater improvements in tinnitus symptoms from bimodal neuromodulation were observed for individuals with greater hyperacusis or tinnitus symptom severity in the TENT-A1 study. Analyses of these subgroups were not prespecified as primary efficacy endpoints in TENT-A1, and therefore, they are included in the primary efficacy endpoints for this TENT-A2 study.

The between-arm analyses will be based on an intention-to-treat estimand tested with multiple regression using enrollment scores as a covariate. Missing data will be handled by using Markov chain Monte Carlo multiple imputation methods [76,77]. The within-arm analyses will be based on a per-protocol estimand and tested with paired two-tailed *t* tests. The use of per-protocol estimand will ensure that the changes in outcome measures within each treatment arm are reflective of real-use scenarios, that is, where the participants use the treatment as directed. The threshold for inclusion in the per-protocol analysis is set at the predefined minimum acceptable compliance threshold previously described.

Additional efficacy analyses will be conducted to evaluate further improvements in the within-arm changes in THI from interim to the end of treatment due to the use of different stimulation settings over time, therapeutic effects in different subtypes of tinnitus participants described previously, and sustained effects by analyzing changes in efficacy outcome measures from the end of treatment to the three follow-up assessments (ie, at 18, 38, and 64 weeks after device fitting). Similar assessments performed for THI will be performed for TFI as additional analyses.

As shown in Figure 2, there are specific hypotheses that will be tested in this study and controlled at an overall significance level of .05. However, as listed in Table 1, several different stimulation settings have been included with the intent of comparing treatment effects between settings and across different time points. Due to limited resources, it is not possible to recruit enough participants to test all of our desired hypotheses or questions. Nevertheless, we will still analyze the data to identify trends that may be further evaluated in a follow-up confirmatory clinical trial. Several key aims that have been purposely incorporated into the design of the study are as follows: (1) to confirm similar within-arm changes in THI and TFI for PS1 in the TENT-A2 study, as observed in the TENT-A1 study for the first 6-week period; (2) to investigate the effect of changing the stimulation settings from PS1 to PS4 in Arm 1, in which a plateau effect was observed after the first 6-week period in the TENT-A1 study when using the same stimulation setting (ie, PS1) for the entire 12-week period; (3) to assess if bimodal stimulation with specific or complex pure tones (without wideband noise) is sufficient to drive therapeutic effects or if inclusion of wideband noise (or wideband noise alone) is required for improving tinnitus symptom severity with bimodal stimulation in Arm 2 and Arm 3; (4) to investigate if bimodal stimulation achieves greater improvements in tinnitus symptom severity compared to acoustic stimulation alone; and (5) to assess the long-term therapeutic effects (up to 12 months after end of treatment) of different stimulation settings across treatment arms and in comparison with the sustained effects observed in the TENT-A1 study. All four treatment arms are considered to be blinded to the participants, because each participant is informed that they are receiving a bimodal neuromodulation treatment during a 12-week period and they do not know which treatment arm consists of the most effective stimulation settings.

Safety analyses will be performed by evaluating the incidence and expectedness of AEs, classified as treatment or nontreatment related and further subclassified according to severity. AEs will be recorded proactively by monitoring significant deteriorations in THI, TFI, MML, hearing thresholds, and oral health and reactively by documenting any AEs reported by participants during the study. All AEs will be analyzed for trends.

Efficacy and safety data analyses will be conducted in compliance with the Consolidated Standards of Reporting Trials guidelines for randomized trials [78].

**Figure:**
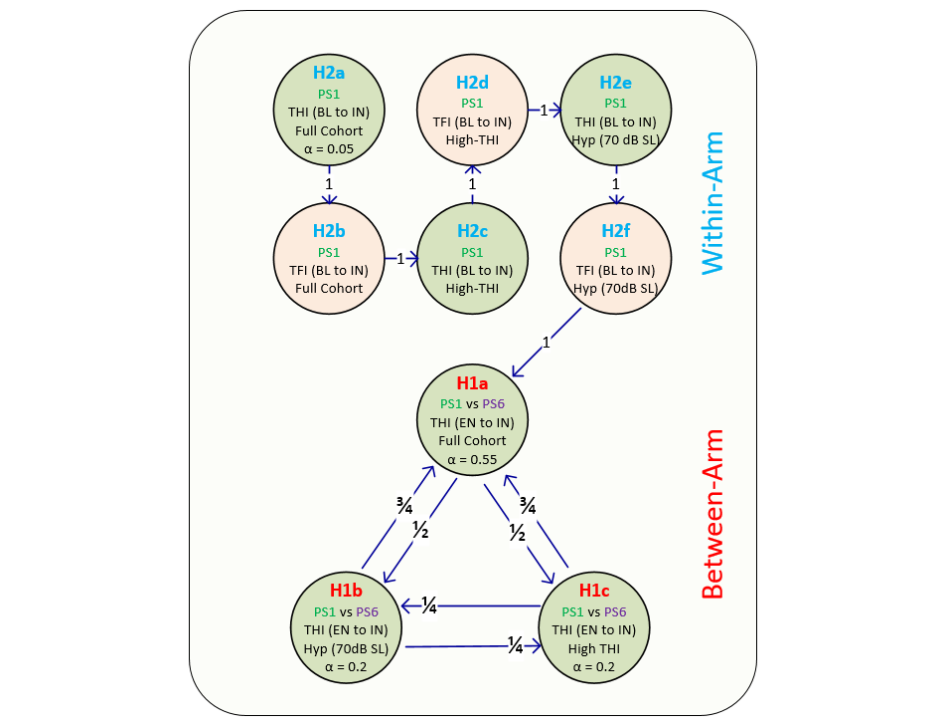
Hypothesis testing accounting for multiple comparisons. The primary endpoints for the TENT-A2 study correspond to several parallel and serial hypotheses depicted in the figure. A *P* value of .05 is initially distributed across four hypotheses (H1a, H1b, H1c, H2a), in which the portion of the *P* value attributed to each hypothesis is indicated by the alpha value. For example, alpha equals 0.55 for H1a, which corresponds to the null hypothesis being rejected if *P*<.0275 (.55x.05). The null hypothesis for H1a is that there is no between-arm difference in changes in mean Tinnitus Functional Index score from enrollment to interim (6-week timepoint) between parameter setting (PS) 1 and PS6 for the full cohort of participants. The null hypothesis for H1b and H1c is that there is no between-arm difference in changes in mean THI score from enrollment to interim between PS1 and PS6 for the hyperacusis subgroup and high tinnitus symptom severity subgroup, respectively. Both are rejected if *P*<.01 (.2x.05). The null hypothesis for H2a is that there is no within-arm change in THI from baseline (average of screening and enrolment scores) to interim for the full cohort of participants. H2a is rejected if *P*<.0025 (.05x.05). Note that the remaining hypotheses (H2b, H2c, H2d, H2e, H2f) can only be tested if the previous hypothesis in the series is rejected. For example, if H2a is rejected, then its portion of the *P* value (*P*=.0025) is transferred to H2b for testing. If H2b is rejected, then its portion of the *P*> value (*P*=.0025) is transferred to H2c, and so on. Similarly, the arrows shown for the between-arm comparisons indicate that if any of the other hypotheses (for H1a, H1b, or H1c) are successfully rejected, then their portion of the *P* value is distributed to its neighbors based on the proportion labeled on each arrow. The null hypothesis for the within-arm comparisons (H2a to H2f) is that there is no within-arm change in THI or Tinnitus Functional Index from baseline to interim for the full cohort of participants, hyperacusis subgroup, or high tinnitus symptom severity subgroup. Note that all within-arm comparisons will be based on a two-sided paired (dependent) t test, while all between-arm comparisons will be based on a linear regression with independent variables of treatment arm and THI score at enrollment. Further details on the statistical analysis plan are provided in the Statistical Methods section. BL: baseline, EN: enrollment, IN: interim, Hyp: hyperacusis subgroup (loudness discomfort level <70 dB sensation level at 500 Hz at screening), High-THI: high tinnitus symptom severity subgroup (THI >56 points at screening); TFI: Tinnitus Functional Index; THI: Tinnitus Handicap Inventory.

## Results

The protocol was independently reviewed and approved by the joint Research Ethics Committee of the Tallaght University Hospital - St James’s Hospital (reference: 2018-03-List 9). The trial was initially registered in ClinicalTrials.gov on May 8, 2018 (identifier: NCT03530306). The first participant was enrolled on March 20, 2018, with the last assessment planned for August 2019. The database is expected to be locked by February 2020, and the data analysis and manuscript submission are expected to be conducted in autumn of 2020. The findings will be distributed to relevant scientific, academic, clinical, health services and participant communities through publications in peer-reviewed and high-impact scientific journals as well as via seminars and talks at conferences.

## Discussion

### Overview

This paper outlines the protocol for a prospective single-site, parallel-arm, randomized, double-blind, comparative study designed to confirm the safety, efficacy, and tolerability of bimodal neuromodulation for tinnitus treatment observed in the previous TENT-A1 trial as well as to determine the therapeutic effects of adjusting the treatment stimulation settings over time and to identify responsive subtypes of tinnitus participants.

This study is important for the tinnitus field for several reasons. First, the findings in TENT-A2 can be compared to those obtained in TENT-A1 in order to assess if the safety and efficacy of bimodal neuromodulation treatment for tinnitus can be confirmed. Replication of clinical trial results is critically needed to build confidence in a field that is currently plagued with skepticism toward new types of treatment methods. Second, there are still only a few large-scale, blinded, randomized clinical trials for tinnitus treatment in which low-quality clinical trial design and reporting have been identified as a major barrier to developing effective therapies [58,79,80]. This study will not only provide valuable insight into the safety and efficacy for different parameter settings of bimodal neuromodulation for tinnitus participants but will also contribute to the establishment of higher clinical standards for evaluating different tinnitus treatments than are currently practiced. Third, there is a movement in the clinical realm toward personalized medicine and optimizing treatments per patient. The design of this study may reveal specific stimulation features and temporal effects of treatment for driving greater improvements in tinnitus in different subtypes of patients and will help move the field toward more reliable treatment outcomes.

### Strengths and Limitations

The main strength of this study is that it is a large, double-blind, randomized clinical trial designed to confirm the safety, efficacy, and tolerability of treatment demonstrated in a previous large, double-blind, randomized clinical trial. Building on the previous trial, this study will further inform our understanding of the contribution or necessity of different sound and tongue stimulation parameters on the clinical efficacy of bimodal stimulation for tinnitus treatment. This study will comprehensively assess the therapeutic effect of different stimulation parameters in predefined patient subgroups that will refine candidature and improve personalization for the intervention in tinnitus patients, in which there are very few large-scale treatment studies providing such subtyping data in the tinnitus field.

A limitation of the study design is that the efficacy due to stimulation settings used during the second 6 weeks of treatment may not be directly comparable with efficacy due to the stimulation settings in the first 6 weeks of treatment because of possible carry-over effects. The cumulative effects from both stimulation settings used in each treatment arm can still be compared between arms to achieve one of the main objectives of the study.

## Abbreviations

CE: Conformité Européenne
CGI: Clinical Global Impression
HL: Hearing Level
MMSE: Mini-Mental State Examination
MML: minimum masking level
STAI: State-Trait Anxiety Inventory
TENT-A1: Treatment Evaluation of Neuromodulation for Tinnitus - Stage A1
TENT-A2: Treatment Evaluation of Neuromodulation for Tinnitus - Stage A2
TFI: Tinnitus Functional Index
THI: Tinnitus Handicap Inventory
TLM: tinnitus loudness matching
UIC: unique identifier code
VAS: visual analogue scale
